# Concomitant occurrence of genetically distinct Hodgkin lymphoma and primary mediastinal lymphoma

**DOI:** 10.1002/ccr3.4504

**Published:** 2021-08-16

**Authors:** Sydney Dubois, Philippe Ruminy, Elodie Bohers, Pierre‐Julien Viailly, Liana Veresezan, Jean‐Michel Picquenot, Victor Bobée, Mathieu Viennot, Dominique Penther, Vincent Camus, Catherine Thieblemont, Camille Pouaty, Hervé Tilly, Fabrice Jardin

**Affiliations:** ^1^ Department of Clinical Hematology Centre Henri Becquerel, INSERM U1245 Rouen France; ^2^ CHU Charles Nicolle Rouen France; ^3^ Hôpital Saint‐Louis Paris France

**Keywords:** Hodgkin lymphoma, molecular characterization, primary mediastinal lymphoma

## Abstract

Synchronous Hodgkin Lymphoma and Primary Mediastinal B‐cell Lymphoma is possible, with molecular analyses proving the absence of clonal filiation between both entities. This suggests a common etiology but the existence of two divergent clones.

## Graphical abstract

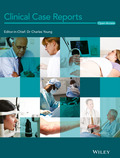

## 1 INTRODUCTION

We describe the case of a patient presenting synchronous Hodgkin Lymphoma and Primary Mediastinal B‐cell Lymphoma. Molecular analyses suggested that both entities arose from two distinct clones. This case highlights the importance of thoroughly characterizing lymphoma disease to best tailor treatment.

The biological proximity between classical Hodgkin Lymphoma (cHL) and Primary Mediastinal B‐cell Lymphoma (PMBL) has been widely described.^1^ Clinically, both diseases present in young adults and frequently present as an anterior mediastinal mass with or without supraclavicular or infraclavicular lymphadenopathies. Distinguishing the two entities on a histological level is crucial, given the divergent therapeutic approaches. Cases of secondary Non‐Hodgkin Lymphoma (NHL) presenting after cHL have been described, with the incidence varying between 1 and 5.9% depending on the series.^2^ Gray zone lymphoma was used as a term for the first time in 1998 to describe a transitional entity between cHL and PMBL, highlighting the existence of overlap, both biological and clinical, between the two diseases.^3^

We describe, herein, the case of a patient who was initially diagnosed with cHL and was found to present synchronous PMBL. Molecular analyses were able to show the absence of clonal filiation between the patient's two lymphomas, suggesting a common etiology but the existence of two distinct and divergent clones.

## 2 CASE PRESENTATION

A 22‐year‐old man presented in January 2018 with a 6‐month history of weight loss, fatigue, night fever, and right cervical lymphadenopathy. Examination revealed additional supraclavicular and bilateral axillary lymphadenopathies. The white blood count (WBC) was 10 600/mm^3^ with 8.3% lymphocytes. Hemoglobin was 13.4g/dL with a normal platelet count. Albumin was normal at 42g/L, Lactate Dehydrogenase (LDH) was normal at 351 U/L, beta2 microglobulin was decreased at 2.2mg/L, and sedimentation rate was increased (62 mm/H).

**FIGURE 1:**
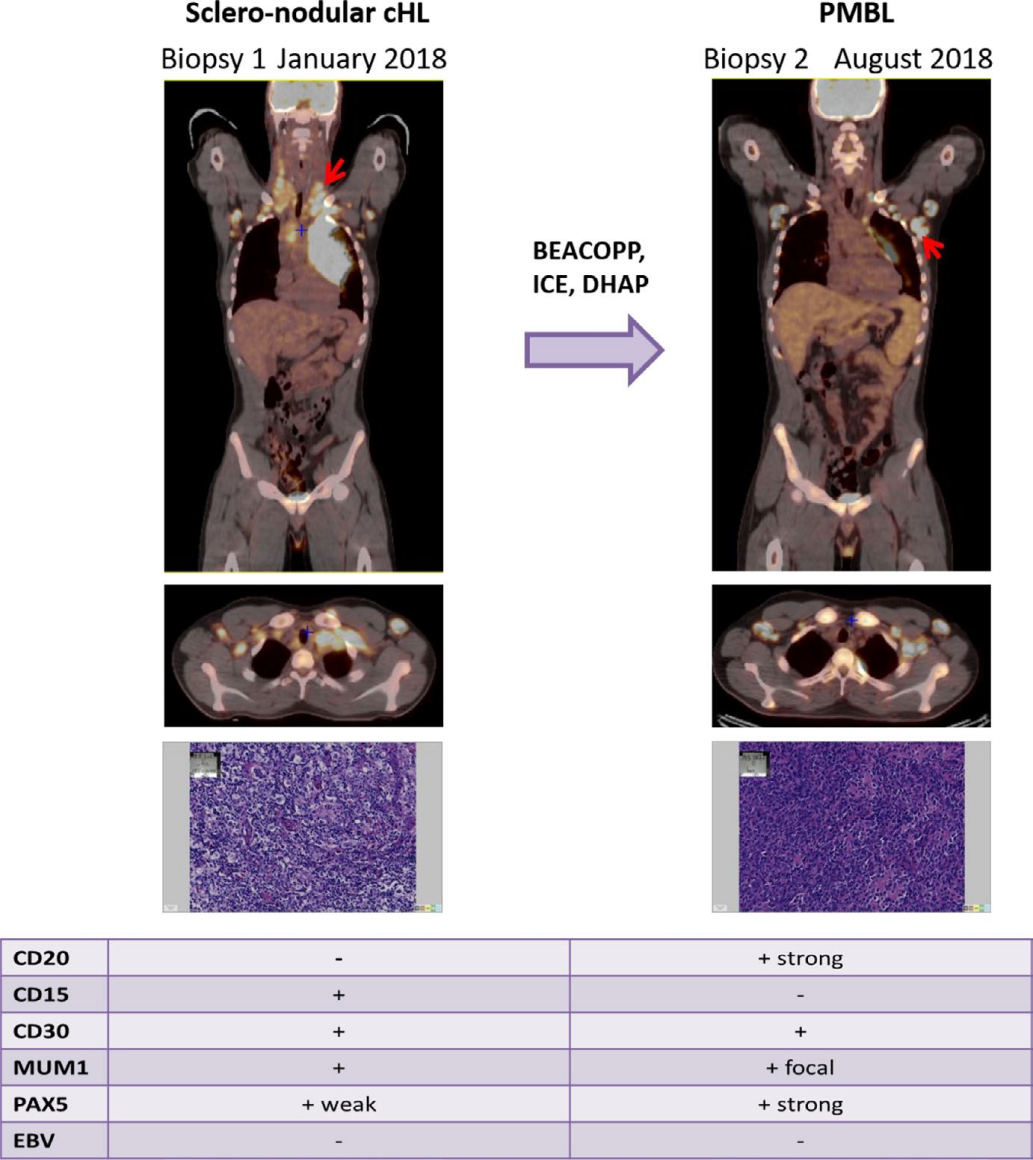
PET‐CT and immunohistochemistry of both synchronous lymphoma entities. PET‐CT performed at specified dates: the red arrow indicates biopsy site. The Hematoxylin Eosin Saffron (HES) stain and the immunohistochemistry results (‐ for negative stain, + for positive stain) of each biopsy are presented underneath.

A biopsy of the right cervical lymphadenopathy performed in January 2018 revealed tumoral proliferation of nodular architecture with certain cells presenting typical aspects of Reed Sternberg cells. Tumoral cells were CD20 negative, CD5 negative, CD30 positive, CD15 positive, MUM1 positive, and weakly positive for PAX5. EBV detection by in‐situ hybridization was negative. Altogether, the morphological and immunophenotypical characteristics of the biopsy were in favor of nodular sclerosis cHL (Figure 1).

The karyotype was normal. Extension was assessed by Positron Emission Tomography‐Computed Tomography (PET‐CT) and identified stage IV cHL with bulky mediastinal mass and invasion of the ribs and pleura. Hasenclever International Prognostic Score (IPS) was 2, with male sex and stage IV as pejorative prognostic markers.

### 2.1 Treatment and outcome

First‐line treatment by BEACOPP (Bleomycin, Etoposide, Adriamycin, Cyclophosphamide, Oncovin, Prednisone, Procarbazine) was proposed according to the AHL 2011 trial.^4^ PET‐CT evaluation following 2 BEACOPP cycles showed partial response with a Deauville score of 5, leading to two additional BEACOPP cycles. Unfortunately, PET‐CT evaluation following 4 BEACOPP cycles showed a dissociated metabolic response as the patient presented continued partial response of the left pleuro‐pulmonary lesion and left axillary lymphadenopathies contrasting with an increase in the intensity of Fluoro‐D‐Glucose (FDG) uptake of the anterior mediastinal mass (Deauville score 4).

At this time, a mediastinal biopsy was deemed unfeasible and salvage treatment with Brentuximab Vedotin (BV) ‐ Ifosfamide, Carboplatin, Etoposide (ICE) was initiated, with the goal of autologous stem‐cell transplantation as consolidation therapy.^5^ After a single cycle of BV‐ICE, clinical and metabolic progression was observed with the appearance of a new left axillary lymphadenopathy. An attempt at ultrasound‐guided biopsy of the new lesion failed in June 2018. Nevertheless, given the patient's rapid clinical deterioration and assumed probable progression of the initial disease, third‐line salvage treatment with Dexamethasone, High dose Aracytine cisPlatine (DHAP) was initiated without new histological evidence. Unfortunately, B symptoms reappeared, and metabolic progression following two cycles of DHAP was confirmed (Deauville score 5). The PET‐CT showed a mediastinal mass but also supraclavicular, infraclavicular and pleural involvement (Figure 1).

Left axillary lymphadenectomy was performed in August 2018 and revealed tumoral cells with strong CD20 positivity, high Ki67 at 70% expression, focal positivity for MUM1, strong PAX5 positivity, CD30 positivity in 50% of tumoral cells, and negativity for CD5, CD3, CD10, and CD15. EBV detection by in‐situ hybridization was negative. Altogether, the clinical, morphological, and immunophenotypical characteristics of the biopsy were in favor of PMBL. The biopsy karyotype identified a complex clone with t(2;3)(p12;q27), add(9)(p12), and MYC rearrangement, confirmed by Fluorescent in‐situ Hybridization (FISH). Both of these features are in favor of PMBL diagnosis.^6^, ^7^ The patient was refractory to Rituximab‐Doxorubicine, Cyclophosphamide, Vindesine, Bleomycin, Prednisone (R‐ACVBP). Anti‐CD19 CAR T‐cell (axicabtagene ciloleucel) therapy was attempted but the patient ultimately succumbed to his disease 16 months after the initial cHL diagnosis.

### 2.2 Molecular investigations

Molecular analyses by both next‐generation sequencing (NGS) and RT‐MLPA^8^ were performed on both biopsies (biopsy 1: cHL in January 2018 and biopsy 2: PMBL in August 2018). Biopsy 1 presented only two *B2 M* mutations, compatible with cHL diagnosis (Table 1). Biopsy 2 presented multiple alterations characteristic of PMBL mutational profiles, notably targeting *STAT6*, *SOCS1*, and *CD58* (Table 1).^9^ There were no common mutations between both biopsies, suggesting no clonal filiation between the two entities. Table S1 details the regions sequenced by the NGS panel for each of the genes specified in Table 1.

**TABLE 1:** Mutational profiles of each biopsy.

Biopsy	Gene	Mutation type	Number of alterations	VAF (%)
1	*B2 M*	frameshift deletion / nonsynonymous SNV	x2	2,1
2	*CD58*	frameshift deletion	x2	15,4–20,3
2	*CDKN2A*	nonsynonymous SNV	x1	14,3
2	*CIITA*	nonsynonymous SNV	x1	32,3
2	*NOTCH1*	nonsynonymous SNV	x1	26,2
2	*PTEN*	nonsynonymous SNV	x1	36,7
2	*SOCS1*	nonsynonymous SNV	x8	37,6–46,4
2	*STAT6*	nonsynonymous SNV	x2	31,5–32,7
2	*TNFAIP3*	frameshift deletion / stopgain	x2	17,2–42,2

Note

Two low VAF (Variant Allele Frequency) *B2 M* mutations were identified in DNA extracted from biopsy 1, compatible with cHL. Mutations characteristic of PMBL were identified in DNA extracted from biopsy 2, notably targeting *CD58*, *SOCS1*, *STAT6*, and *TNFAIP3*.

**FIGURE 2:**
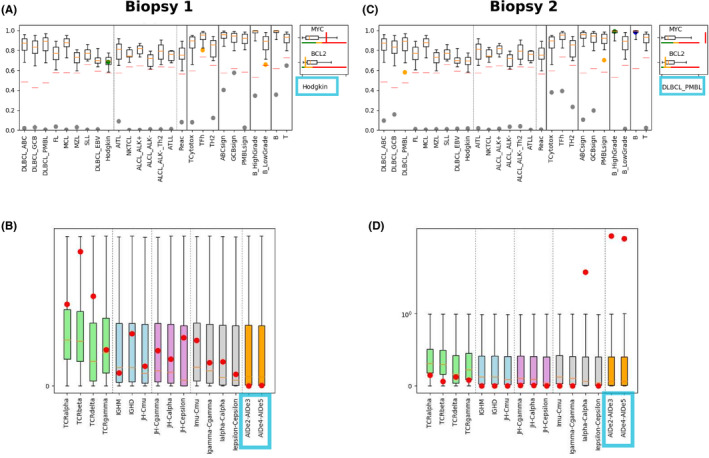
RT‐MLPA profiles of each biopsy. A and C show that RT‐MLPA classifies biopsies 1 and 2 as cHL and PMBL, respectively. B and D show differential expression of certain genes by biopsies 1 and 2, respectively, and notably AID (framed).

RT‐MLPA gene expression profiling (GEP) categorized biopsy 1 as cHL with no expression of activation‐induced cytidine deaminase (AID) (Figure 2A, B). The same technique categorized biopsy 2 as PMBL with very high AID expression (Figure 2C, D). As such, the two biopsies’ gene expression profiles also pointed toward a lack of clonality between the two entities.

**FIGURE 3:**
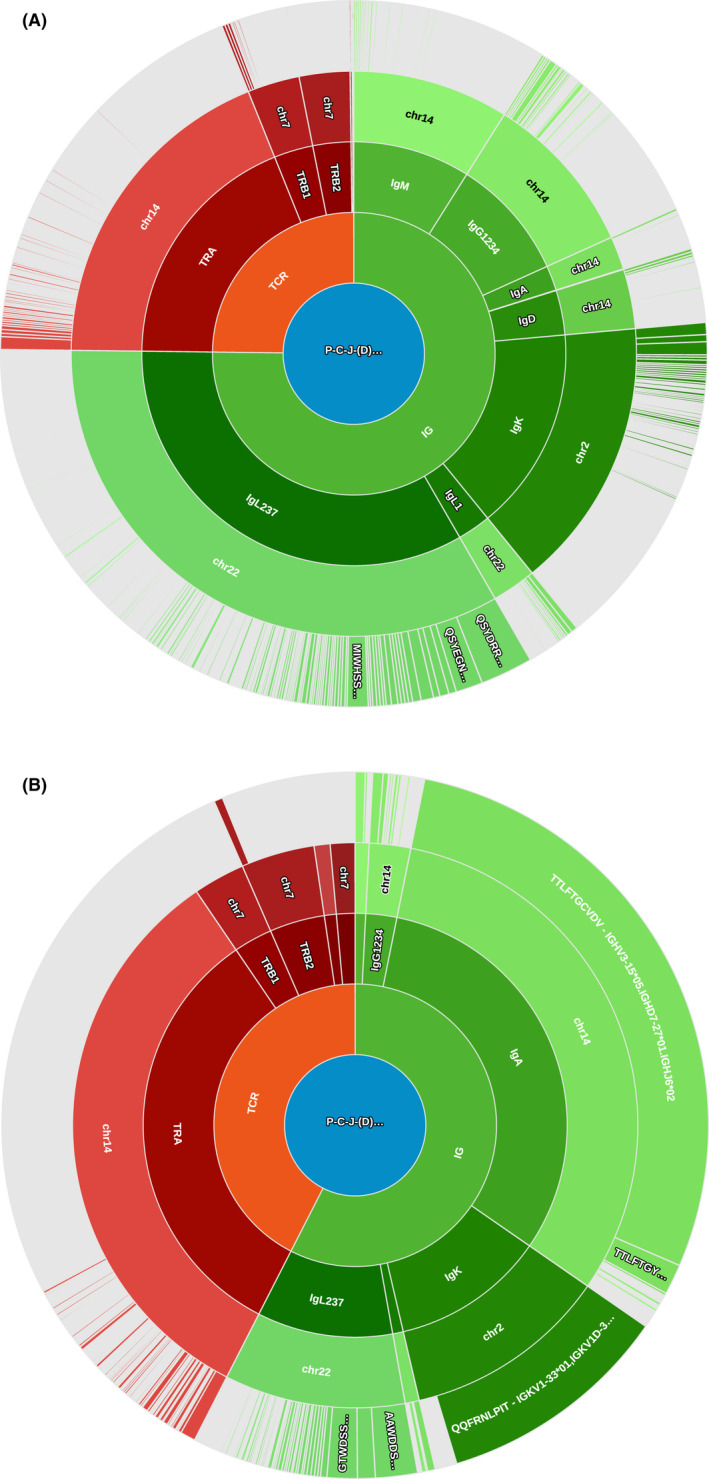
RACE profiles of each biopsy's VDJ population. First inner circle indicates analysis of immunoglobulins (green hues) or TCR (red hues). Second inner circle delimits the exploration of heavy chains (IgM, IgG, IgA, IgD) and light chains (IgK, IgL) regarding immunoglobulin analysis, and the exploration of alpha, beta, gamma, and delta chains regarding TCR analysis. Third inner circle indicates the chromosome involved for each of the previously described chains. The most outer circle exhibits the identified clones: The perimeter occupied by each clone is directly correlated to the number of reads identifying the clone in question. Clones are labeled by the amino‐acid sequence detected. (A) shows the profile of biopsy 1, with no outlier clone. (B) shows the profile of biopsy 2, identifying an IgA/IgK clonality.

Finally, we performed a modified 5’ Rapid amplification of cDNA ends (RACE) technique targeting both heavy and light chains of the BCR as well as TCR alpha, beta, gamma, and delta chains (Figure 3A, B). Using this technique, IgA/Igκ clonality was identified for the PMBL biopsy whereas no specific Ig clonality was identified in the cHL biopsy. Furthermore, the t(2;3) translocation identified by karyotype in the PMBL biopsy was confirmed by this technique, resulting in an Igκ‐LPP transcript (data not shown). The results of this technique, in addition to the differences in gene mutation and gene expression analyses, seem to confirm a different clonal derivation for the two biopsies.

## 3 DISCUSSION

The biological proximity between cHL and PMBL is well‐known^1^ and the possibility of transformation from cHL to PMBL are well‐established.^2^, ^10^ The case described, herein, highlights the impact of molecular analysis in attempting to prove the absence of genetic filiation between synchronous cHL and PMBL. We were not able to perform microdissection of the cHL case in order to definitely prove distinct clonal etiology of the two cases but all molecular analyses performed to suggest the existence of two distinct clones, which share a common early etiological mechanism, explaining their synchronous appearance. Histologically, the persistence of CD30 positivity in both biopsies with the appearance of CD20 positivity only in biopsy 2 and also seems to corroborate this hypothesis. In a previously described case where the patient developed DLBCL at a different site after treatment for cHL,^11^ CD20 expression was in fact positive in the initial cHL biopsy, leading to the possibility that the initial diagnosis should perhaps have been that of gray zone lymphoma.

Distinct chemosensitivity is manifest between the two tumor types, highlighting the importance of a precise diagnosis, with the aid of precise molecular characterization if necessary and possible. Indeed, standard cHL treatment was initiated but rapid progression was observed nonetheless. Lack of patient response to several treatment lines ultimately led to the discovery of synchronous PMBL, which was then unfortunately refractory to R‐ACVBP and CAR T‐cell therapy. The importance of repeating biopsies when faced with progressive disease is illustrated in this case, even when progression of the initial disease would seem to be the most probable scenario.

The capacity of GEP to distinguish PMBL and cHL has already been demonstrated.^12^, ^13^ We reported, herein, a concrete case of using molecular characterization by NGS and GEP to prove the existence of two synchronous and distinct lymphoma entities with no clonal filiation. In this rare presentation, had both entities been diagnosed at baseline, perhaps combination Rituximab‐BV‐chemotherapy^14^ or the addition of checkpoint inhibitors to Rituximab‐chemotherapy treatment would have proved a better option than sequential therapy.

## 4 CONCLUSION

We have reported a case of synchronous cHL and PMBL with molecular analyses of both biopsies suggesting that they arose from two distinct B clones. Given the distinct chemosensitivity of both entities, this case highlights the importance of identifying the existence of potential synchronous cHL and PMBL as soon as possible in order to improve patient prognosis.

## CONFLICT OF INTEREST

The authors declare no conflict of interest with regard to this manuscript.

## AUTHOR CONTRIBUTIONS

SD collected the data and wrote the manuscript, PR performed RT‐MLPA, EB performed NGS, P‐JV performed bioinformatics analysis, LV and J‐MP gave histopathological diagnoses, VB performed RT‐MLPA, MV performed RT‐MLPA and NGS, DP performed karyotype and FISH, VC was in charge of patient clinical management, CT supervised CAR T therapy, CP was in charge of patient clinical management, HT and FJ supervised patient clinical management, molecular analyses, and data collection.

## ETHICAL APPROVAL

The patient gave their written informed consent for all investigations and publication of this case report.

## Supporting information

Table1

## Data Availability

The data that support the findings of this study are available from the corresponding author upon reasonable request.
